# Haplotype‐resolved DNA methylome of African cassava genome

**DOI:** 10.1111/pbi.13955

**Published:** 2022-12-08

**Authors:** Zhenhui Zhong, Suhua Feng, Ben N. Mansfeld, Yunqing Ke, Weihong Qi, Yi‐Wen Lim, Wilhelm Gruissem, Rebecca S. Bart, Steven E. Jacobsen

**Affiliations:** ^1^ Department of Molecular, Cell and Developmental Biology University of California Los Angeles CA USA; ^2^ Eli & Edythe Broad Center of Regenerative Medicine & Stem Cell Research University of California Los Angeles CA USA; ^3^ Donald Danforth Plant Science Center St. Louis MO USA; ^4^ Functional Genomics Center Zurich ETH Zurich and University of Zurich Zurich Switzerland; ^5^ Department of Biology, Institute of Molecular Plant Biology, ETH Zürich Zürich Switzerland; ^6^ Biotechnology Center National Chung Hsing University Taichung City Taiwan; ^7^ Howard Hughes Medical Institute, University of California Los Angeles CA USA

**Keywords:** haplotype‐resolved, DNA methylation, cassava

Cytosine DNA methylation is involved in transposable element (TE) silencing, imprinting and X‐chromosome inactivation. Plant DNA methylation is mediated by MET1 (mammalian DNMT1), DRM2 (mammalian DNMT3) and two plant‐specific DNA methyltransferases, CMT2 and CMT3 (Law and Jacobsen, 2010). *De novo* DNA methylation in plants is established by DRM2 via the plant‐specific RNA‐directed DNA methylation (RdDM) pathway that depends on two DNA‐dependent RNA polymerases, Pol IV and Pol V (Gallego‐Bartolome *et al.,* 2019; Law and Jacobsen, 2010; Stroud *et al.,* 2013). The DNA methylome of cassava has been previously documented based on its haploid collapsed genome (Wang *et al.,* 2015). Since the cassava genome is highly heterozygous, DNA methylome of the haplotype‐collapsed genome misses many features of the methylome. With the development of long‐read sequencing and chromosomal conformation capture techniques, haplotype‐resolved genomes are available for highly heterozygous genomes (Mansfeld *et al.,* 2021; Qi *et al.,* 2022; Sun *et al.,* 2022; Zhou *et al.,* 2020), which provides high‐quality reference genomes facilitating studies of haplotype‐resolved DNA methylomes.

To dissect the haplotype‐resolved DNA methylome of cassava, we conducted methylome studies in two haplotype genome‐resolved accessions of cassava (TME7 and TME204) using whole‐genome bisulfite sequencing (WGBS) and enzymatic methyl‐seq (EM‐seq), respectively (Feng *et al.,* 2020; Mansfeld *et al.,* 2021; Qi *et al.,* 2022). Sequencing reads were mapped to different haplotypes individually allowing zero mismatches and one best hit, which allowed the separation of reads belonging to different haplotypes. Overall, we found that although both WGBS and EM‐seq methods were used, the two haplotypes have similar whole‐genome methylation levels in TME7 and TME204 (Figure 1a; Figure S1). We further plotted methylation levels over transcribed regions of protein‐coding genes and TEs, and observed similar methylation levels between different haplotypes (Figure 1b,c; Figure S2A,B).

**Figure 1:**
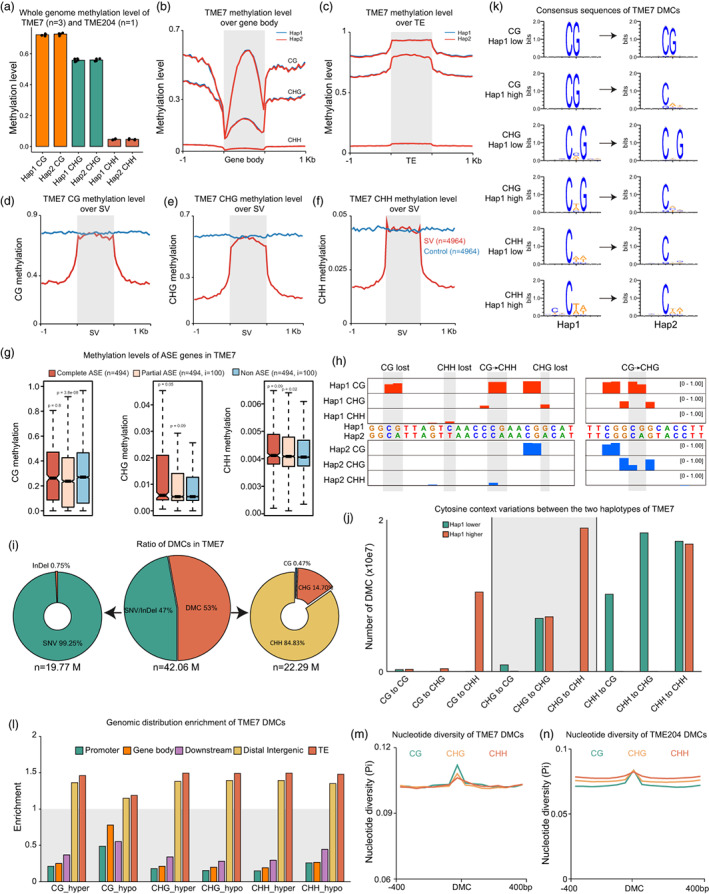
Haplotype‐resolved DNA methylome of African cassava. (a) Whole‐genome methylation levels of hap1 and hap2 haplotypes in TME7 (*n* = 3) and TME204 (*n* = 1). (b) Metaplot of CG, CHG and CHH methylation levels over protein‐coding genes and flanking 1‐kb sequences of hap1 and hap2 haplotypes in TME7. (c) Metaplot of methylation levels over transposon elements and flanking 1 kb sequences in TME7. (d–f) Metaplot of methylation levels over structural variation regions and flanking 1 kb sequences in TME7. Regions of equal length were randomly selected in the genome (blue line). (g) Methylation levels of complete, partial and non‐ASE genes in TME7. (h) Representative screenshots of DMCs. Methylation site losses and mC context changes are indicated on top of the track (red: hap1; blue: hap2). (i) Ratio of DMCs caused by SNP/InDel and DMCs caused by different methylation levels in TME7. (j) Numbers of different types of cytosine context variations between the two haplotypes in TME7. (k) Consensus sequences of DMCs in TME7. (l) Genomic distribution enrichment of DMCs in TME7. (m–n) Nucleotide diversity of 400‐bp flanking regions of DMC in TME7 (m) and TME204 (n). Panels i, j, l and k show results for TME7. Results for TME204 are shown in Figure S2.

Previous studies have revealed large numbers of haplotype‐specific structural variants (SVs) in cassava (Mansfeld *et al.,* 2021; Qi *et al.,* 2022). To understand how DNA methylation is associated with these SVs, we analysed methylation levels of these SVs. Focusing on places where haplotype‐specific SVs occur, we found that flanking regions of SVs have approximately two times lower methylation levels than random controls and SVs, suggesting that SVs preferentially take place at lowly methylated regions, with the SVs turning these regions to heavily methylated regions (Figure 1d–f). The gain of methylation over these SVs may subsequently inactivate surrounding regions and silence nearby genes. Allele‐specific expression (ASE) refers to preferential expression of the allele transcribed from one haplotype (Gaur *et al.,* 2013). Previous study has catalogued ASEs into complete ASEs and partial ASEs (Mansfeld *et al.,* 2021). Interestingly, complete ASE genes showed higher gene body CHG methylation than partial (*P* = 0.05) and non‐ASE genes and partial ASE genes showed lower CG methylation than others (*P* = 3.8 e‐08, Figure 1g), suggesting that DNA methylation alterations between alleles from different haplotypes are frequently accompanied with ASEs.

Next, we analysed differentially methylated cytosines between haplotypes (haplotypic DMCs). We first aligned the two haplotypes and identified syntenic cytosines (Figure S2C). Methylation levels of syntenic cytosines were compared between two haplotypes. We observed at least three scenarios (Figure 1h) that resulted in differential methylations between haplotypes: (i) SNP/InDel in one haplotype leads to the loss of the cytosine (47.17%), among which more than 99.25% are SNP variants (Figure 1i; see TME204 data in Figure S2D); (ii) Cytosine context alterations (e.g. CG in hap1 and CHH in hap2) that lead to the methylation level changes detected in our analysis (28.98%); and (iii) Cytosines stay in the same contexts between haplotypes but exhibit different methylation levels (23.85%). For DMCs caused by scenarios 2 and 3, the majority of them were CHH variations (Figure 1i). When CG sites are mutated into CHG or CHH sites, they are more likely to be hypomethylated, whereas CHH sites are more likely to be hypermethylated when mutated into CHG and CG sites (Figure 1j,k; Figure S2E,F). Furthermore, we found that DMCs are frequently detected at TEs and distal intergenic regions, while depleted at gene bodies and flanking regions, suggesting that TEs are hot spots for frequent DNA methylation changes (Figure 1l, Figure S2G). Finally, we investigated the genetic diversity of DMC sites and 400‐bp flanking sequences and found that nucleotide diversity of DMC sites is significantly higher than that of flanking sequences (Figure 1m,n). Higher nucleotide diversity of DMCs demonstrated that DMC sites are under more frequent natural selection and revealed the crosstalk between sequence variations and DNA methylation mutations. Together, our analyses compared haplotype‐resolved DNA methylomes of cassava and placed genomic heterozygosity within the haplotypic epigenetic regulatory landscape.

## Conflicts of interest

The authors declare no conflict of interest.

## Author contributions

Z.Z., S.F., B.M., W.G., R.B. and S.J. designed the research. Z.Z., W.Q. and Y.K. performed data analysis. Y.L. and S.F. prepared sequencing libraries. Z.Z., S.F. and S.J. wrote the manuscript.

## Supporting information


**Appendix S1** Methods.
**Figure S1** Whole genome methylation over chromosomes of TME204 and TME7.
**Figure S2** Haplotype‐resolved DNA methylome of African cassava.

## Data Availability

The DNA methylome data are available in Gene Expression Omnibus under accession number GSE192748 (https://www.ncbi.nlm.nih.gov/geo).
