# Supplementary material for: Haplotype‐resolved DNA methylome of African cassava genome
Source: Plant Biotechnol J. 2022 Dec 8;21(2):247–9. doi: 10.1111/pbi.13955 (PMC9884013; doi:10.1111/pbi.13955)
Supplement: Supplementary file 1 — Appendix S1 Methods. Figure S1 Whole genome methylation over chromosomes of TME204 and TME7. Figure S2 Haplotype‐resolved DNA methylome of African cassava. [file PBI-21-247-s001.docx]

**Haplotype-resolved DNA methylome of African cassava genome**

Zhenhui Zhong^1^, Suhua Feng^1,2^, Ben N. Mansfeld^3^, Yunqing Ke^1^, Weihong Qi^4^, Yi-Wen Lim^5^, Wilhelm Gruissem^5^, Rebecca S. Bart^3^, Steven E. Jacobsen^1,2,6†^

^1^ Department of Molecular, Cell and Developmental Biology, University of California, Los Angeles, CA 90095, USA

^2^ Eli & Edythe Broad Center of Regenerative Medicine & Stem Cell Research, University of California, Los Angeles, CA 90095, USA

^3^ Donald Danforth Plant Science Center, St. Louis, MO 63132, USA

^4^ Functional Genomics Center Zurich, ETH Zurich and University of Zurich, Winterthurerstrasse 190, 8057 Zurich, Switzerland

^5^ Institute of Molecular Plant Biology, Department of Biology, ETH Zürich, Universitätsstrasse 2, 8092 Zürich, Switzerland

^6^ Howard Hughes Medical Institute, University of California, Los Angeles, CA 90095, USA

† Correspondence to: jacobsen@ucla.edu (S.E.J.)

Keywords: Haplotype-resolved, DNA methylation, Cassava

**Supporting Information**

**Method**

**DNA methylation library preparation**

Methylome of TME7 samples were sequenced by whole genome bisulfite sequencing (WGBS). Briefly, genomic DNA was end-repaired and ligated with TruSeq DNA single-end adapters (Illumina) using a Kapa DNA HyperPrep kit (Roche). Adapter-ligated DNA was converted with an EpiTect Bisulfite Kit (Qiagen). Converted DNA was PCR-amplified by MyTaq polymerase (Bioline) for 12 cycles. Methylome of TME204 sample was sequenced by Enzymatic Methyl-seq (EM-seq). EM-seq libraries were prepared from sheared DNA using an Enzymatic Methyl-seq kit following manufacturer instructions (New England BioLabs) with 6 cycles of PCR (Feng et al., 2020). The libraries were run on a D1000 ScreenTape (Agilent) to determine the quality and size, and then purified by AMPure XP beads (Beckman Coulter). Library concentrations were measured with a Qubit dsDNA Broad-Range Assay kit (ThermoFisher). Libraries were sequenced on a HiSeq 2500 or NovaSeq 6000 sequencer (Illumina). We have used two methods, WGBS and EM-seq, for TME7 and TME204, respectively. In our previous study (Feng et al., 2020), we found that these two methods have different performance when the input DNA or the amplification cycle numbers are different, while their performance is similar under most suitable conditions as used here.

**Methylome mapping**

WGBS and EM-seq reads were mapped to haplotype 1 and haplotype 2 genomes of TME7 (Mansfeld et al., 2021) or TME204 (Qi et al., 2022) by Bsmap (v2.90) allowing 0 mismatches and 1 best hit (-v 0 -w 1) (Xi & Li, 2009). Duplicated reads were removed with SAMtools (v1.3.1) (Li et al., 2009). Reads with three or more consecutive methylated CHH sites were considered as unconverted reads and removed in the subsequent analyses. Conversion rate was estimated by calculating methylation level of the chloroplast genome. DNA methylation level at each cytosine was calculated by number of methylated C vs. total C and T account. To call Differentially Methylated Cytosines (DMCs), two haplotype genomes were aligned with each other by LastZ with script published previously (Zhou et al., 2020). Only cytosines with at least 3000 bp syntenic flanking regions were kept in the analysis. Two-tailed Fisher’s exact test was used to calculate p-value between syntenic cytosines. We used methylation differences > 0.4 for CG, > 0.2 for CHG, and > 0.1 for CHH, and p-value < 0.05 as cutoff, to define DMCs. Methylation track files were visualized with Integrative Genomics Viewer (IGV, v3.0) (Robinson et al., 2011). Structural Variations (SVs) and Allele Specific Expressions (ASEs) information are from previous studies (Mansfeld et al., 2021; Qi et al., 2022). Only insertion SVs at haplotype 1 genome have been used for methylation metaplot since the counterpart in the other haplotype is the same with the flanking 1000 bp regions. Complete and partial ASE genes are defined in the previous publication (Mansfeld et al., 2021). Briefly, “complete ASE” is defined if significant ASE was detected and the log2(fold-change) between two alleles was >5. The “partial ASE” is defined if statistically significant ASE was observed, but the allelic log2(fold-change) did not pass the threshold of 5. We have only presented DNA methylation level of allele with higher expression in haplotype 1. Since the number of partial ASEs and non-ASE controls are much higher than that of complete ASEs, we selected an equal number of complete ASEs and partial ASEs or non-ASE controls with 100 times random iterations.

**Consensus sequence, DMC enrichment, and nucleotide diversity analysis**

Sequence consensus analysis was performed with WebLogo (v3.6.0) (Crooks et al., 2004). Flanking 4 bp sequences of DMC were obtained with bedtools (v2.26.0) getfasta function and used as input sequences of WebLogo (Quinlan & Hall, 2010). Genomic distribution enrichment analysis was conducted with ChIPseeker and bedtools (Yu et al., 2015). DMC sites and random control sites of equal number were used as input of ChIPseeker. For transposable element (TE) enrichment analysis, DMC sites and random control sites of equal number were intersected with TE regions to calculate sites that distributed within or outside TE regions. For nucleotide diversity analysis, DMC sites and flanking 400 bp regions were used as input of Pim (v0.3) with 50 bp window length (-w) and 50 bp steps between windows (-S) (Haubold et al., 2011).


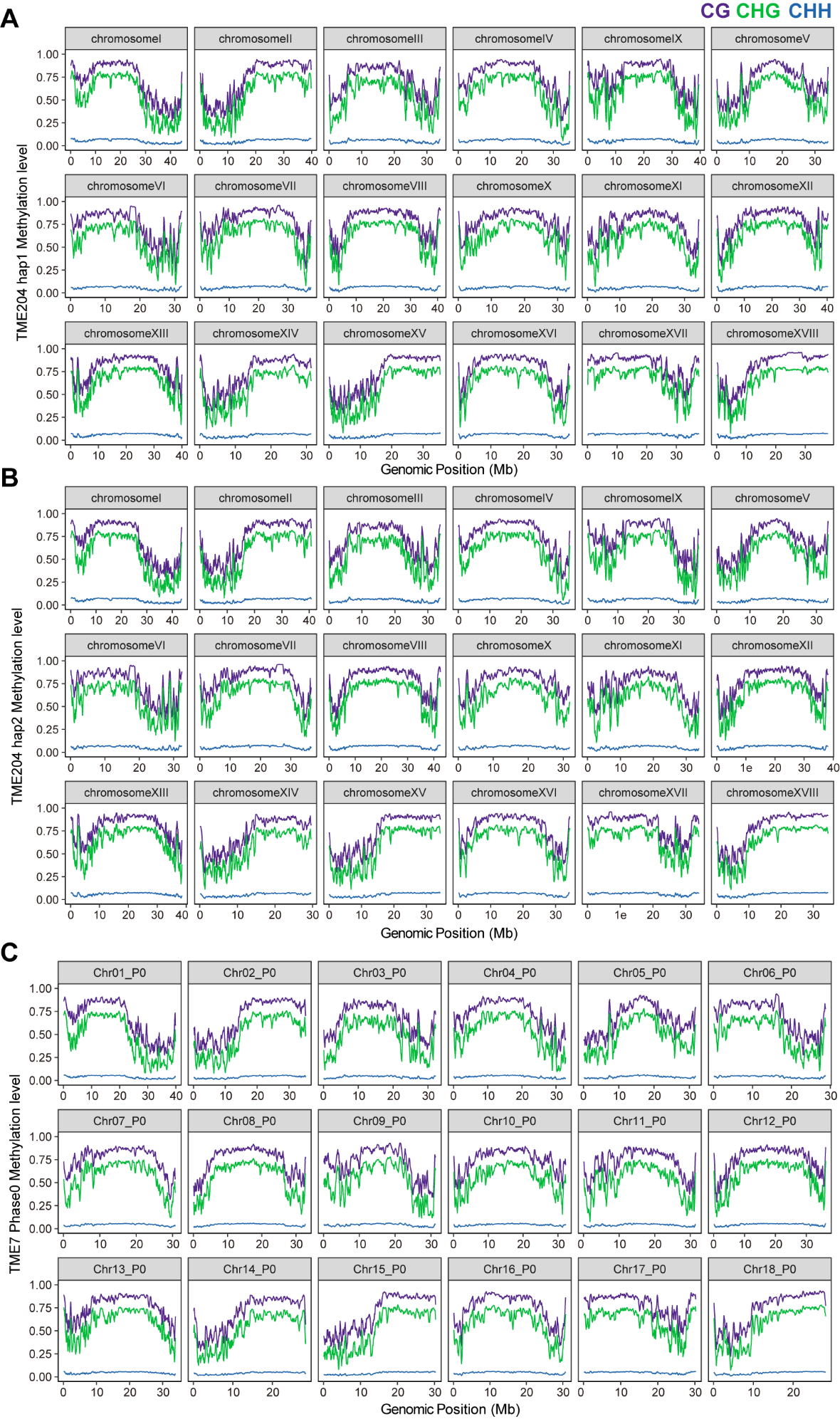


**Supplemental Figure 1. Whole genome methylation over chromosomes of TME204 and TME7.**


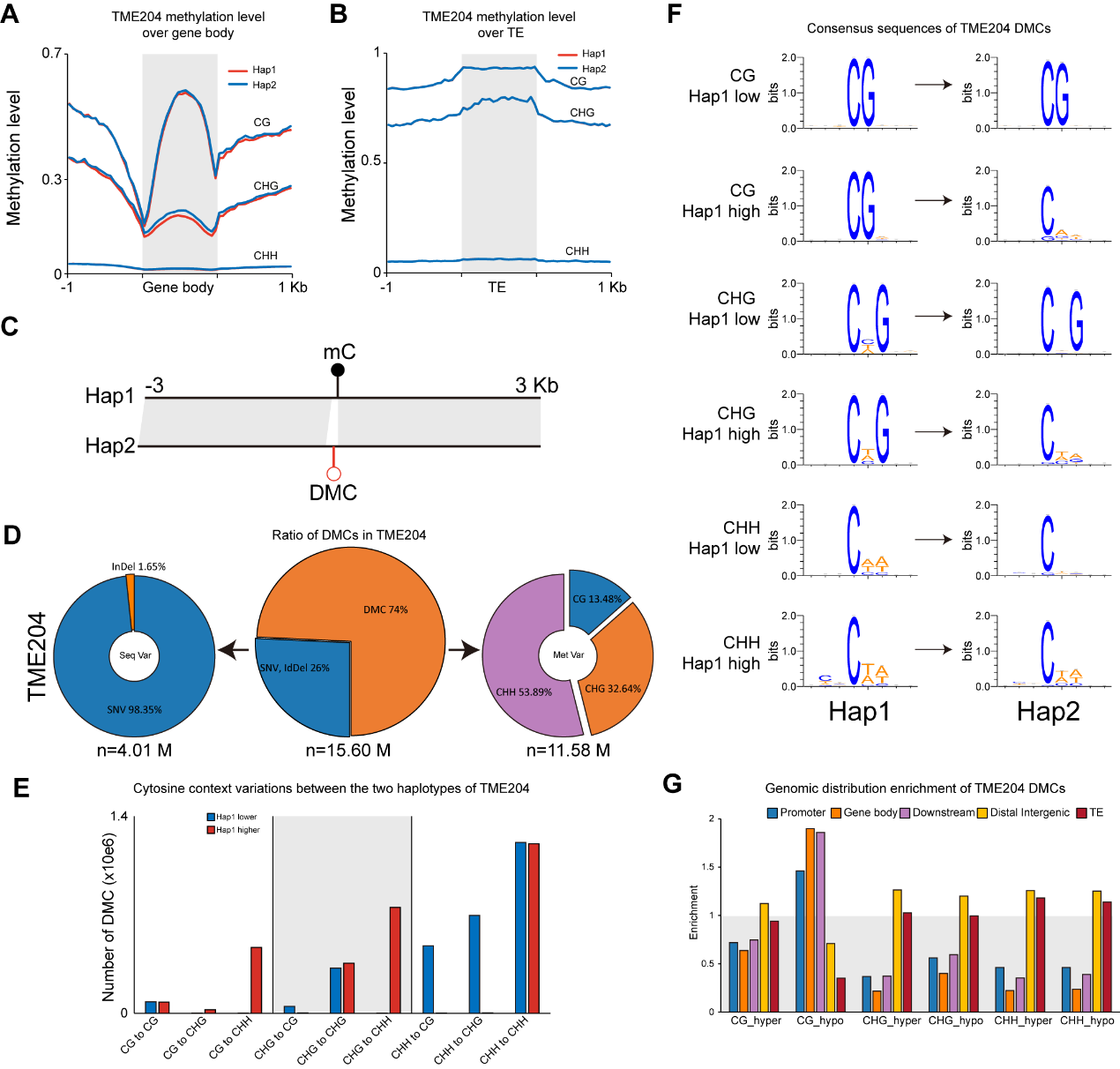


**Supplemental Figure 2 Haplotype-resolved DNA methylome of African cassava.** (A) Metaplot of CG, CHG, and CHH methylation levels over protein-coding genes and flanking 1Kb sequences of hap1 and hap2 haplotypes in TME204. (B) Metaplot of CG, CHG, and CHH methylation levels over transposon elements and flanking 1Kb sequences in TME204. (C) Schematic diagram showing identification of differentially methylated cytosine (DMC). Only cytosines with > 3Kb flanking sequences aligned between the two haplotypes are analyzed. (D) Ratio of DMCs caused by SNP/InDel, and DMCs caused by different methylation levels (differences in CG > 0.4, in CHG > 0.2, and in CHH > 0.1) in TME204. (E) Numbers of different types of cytosine context variations between the two haplotypes of TME204. (F) Consensus sequences of DMCs of TME204. (G) Genomic distribution enrichment of DMCs of TME204.

**Reference**

Crooks, G. E., Hon, G., Chandonia, J.-M., & Brenner, S. E. (2004). WebLogo: a sequence logo generator. *Genome Research*, *14*(6), 1188–1190.

Feng, S., Zhong, Z., Wang, M., & Jacobsen, S. E. (2020). Efficient and accurate determination of genome-wide DNA methylation patterns in Arabidopsis thaliana with enzymatic methyl sequencing. *Epigenetics and Chromatin*, *13*(1). https://doi.org/10.1186/s13072-020-00361-9

Haubold, B., Reed, F. A., & Pfaffelhuber, P. (2011). Alignment-free estimation of nucleotide diversity. *Bioinformatics*, *27*(4), 449–455.

Li, H., Handsaker, B., Wysoker, A., Fennell, T., Ruan, J., Homer, N., Marth, G., Abecasis, G., Durbin, R., & Genome Project Data Processing, S. (2009). The Sequence Alignment/Map format and SAMtools. *Bioinformatics*, *25*(16), 2078–2079. https://doi.org/10.1093/bioinformatics/btp352

Mansfeld, B. N., Boyher, A., Berry, J. C., Wilson, M., Ou, S., Polydore, S., Michael, T. P., Fahlgren, N., & Bart, R. S. (2021). Large structural variations in the haplotype‐resolved African cassava genome. *The Plant Journal*, *108*(6), 1830–1848.

Qi, W., Lim, Y.-W., Patrignani, A., Schläpfer, P., Bratus-Neuenschwander, A., Grüter, S., Chanez, C., Rodde, N., Prat, E., & Vautrin, S. (2022). The haplotype-resolved chromosome pairs of a heterozygous diploid African cassava cultivar reveal novel pan-genome and allele-specific transcriptome features. *GigaScience*, *11*.

Quinlan, A. R., & Hall, I. M. (2010). BEDTools: a flexible suite of utilities for comparing genomic features. *Bioinformatics*, *26*(6), 841–842.

Robinson, J. T., Thorvaldsdóttir, H., Winckler, W., Guttman, M., Lander, E. S., Getz, G., & Mesirov, J. P. (2011). Integrative genomics viewer. *Nature Biotechnology*, *29*(1), 24–26.

Xi, Y., & Li, W. (2009). BSMAP: whole genome bisulfite sequence MAPping program. *BMC Bioinformatics*, *10*(1), 232.

Yu, G., Wang, L.-G., & He, Q.-Y. (2015). ChIPseeker: an R/Bioconductor package for ChIP peak annotation, comparison and visualization. *Bioinformatics*, *31*(14), 2382–2383.

Zhou, Q., Tang, D., Huang, W., Yang, Z., Zhang, Y., Hamilton, J. P., Visser, R. G. F., Bachem, C. W. B., Robin Buell, C., & Zhang, Z. (2020). Haplotype-resolved genome analyses of a heterozygous diploid potato. *Nat Genet*, *52*(10), 1018–1023.
